# Transcriptome profiling of skeletal muscles from Korean patients with Bethlem myopathy

**DOI:** 10.1097/MD.0000000000033122

**Published:** 2023-03-03

**Authors:** Seung-Ah Lee, Ji-Man Hong, Jung Hwan Lee, Young-Chul Choi, Hyung Jun Park

**Affiliations:** a Department of Neurology, Ewha Womans University Mokdong Hospital, Ewha Womans University College of Medicine, Yangcheon-gu, Seoul, Republic of Korea; b Department of Neurology, Yongin Severance Hospital, Yonsei University College of Medicine, Yongin, Gyeonggi-do, Republic of Korea; c Department of Neurology, Seoul St. Mary’s Hospital, College of Medicine, Seocho-gu, Seoul, Republic of Korea; d Department of Neurology, Gangnam Severance Hospital, Yonsei University College of Medicine, Seoul, Republic of Korea.

**Keywords:** Bethlem myopathy, COL6A1, COL6A2, RNA-seq, transcriptome

## Abstract

Bethlem myopathy is one of the collagens VI-related muscular dystrophies caused by mutations in the collagen VI genes. The study was designed to analyze the gene expression profiles in the skeletal muscle of patients with Bethlem myopathy. Six skeletal muscle samples from 3 patients with Bethlem myopathy and 3 control subjects were analyzed by RNA-sequencing. 187 transcripts were significantly differentially expressed, with 157 upregulated and 30 downregulated transcripts in the Bethlem group. Particularly, 1 (microRNA-133b) was considerably upregulated, and 4 long intergenic non-protein coding RNAs, LINC01854, MBNL1-AS1, LINC02609, and LOC728975, were significantly downregulated. We categorized differentially expressed gene using Gene Ontology and showed that Bethlem myopathy is strongly associated with the organization of extracellular matrix (ECM). Kyoto Encyclopedia of Genes and Genomes pathway enrichment reflected themes with significant enrichment of the ECM-receptor interaction (hsa04512), complement and coagulation cascades (hsa04610), and focal adhesion (hsa04510). We confirmed that Bethlem myopathy is strongly associated with the organization of ECM and the wound healing process. Our results demonstrate transcriptome profiling of Bethlem myopathy, and provide new insights into the path mechanism of Bethlem myopathy associated with non-protein coding RNAs.

## 1. Introduction

Collagen VI-related myopathy is a group of genetic disorders that affects skeletal muscles and connective tissues. It is clinically characterized by proximal muscle weakness, joint contractures, and distal joint hyperlaxity.^[1,2]^ Inheritance patterns can also be autosomal dominant or recessive.^[1]^ The severity of the clinical symptoms varies from the milder Bethlem myopathy to the more severe Ullrich congenital muscular dystrophy. Ullrich congenital muscular dystrophy showed congenital muscle weakness, hypotonia, striking joint hyperlaxity, particularly of the distal joints, in conjunction with contractures of the proximal joints, including the hips, elbows, and spine.^[3]^ In Bethlem myopathy, the symptom onset may also be congenital; however, hypotonia at birth is usually rare.^[4]^ Children with Bethlem myopathy have mild muscle weakness and contractures, including in the ankle dorsiflexors and long finger flexors. Collagen VI-related myopathy is caused by the pathogenic variants of *COL6A1, COL6A2*, and *COL6A3*. Three genes encode the α1, α2, and α3 chains of collagen VI, which are major components of the muscle extracellular matrix (ECM) forming a microfibrillar network and basement membrane.^[5,6]^ The 3 collagen VI alpha chains assemble into tetramers before being secreted into the ECM. Pathogenic variants in any one of the 3 collagen VI genes result in either the loss or misfolding of collagen VI in the muscle ECM.^[1]^ However, the exact mechanism by which collagen VI deficiency or misfolding in the muscle ECM leads to myopathy is not fully understood.

Microarray analysis and RNA-sequencing (RNA-seq) are useful gene expression platforms, which show molecular changes in biological samples and define the biological pathways involved in disease pathogenesis.^[7,8]^ Several studies have analyzed gene expression profiles using microarray analysis and RNA-seq in muscles or cells of patients and mouse models with collagen VI-related myopathy.^[9–11]^ Collagen VI-related myopathy has been reported several times in Korea.^[12–16]^ Bethlem myopathy, a mild phenotype of collagen VI-related myopathy, is the most common cause of congenital myopathy in Korea.^[13]^

To identify the pathogenic mechanism of Bethlem myopathy, we comprehensively analyzed the gene expression profiles in the skeletal muscles of patients with Bethlem myopathy using RNA-seq.

## 2. Materials and Methods

### 2.1. Study subjects

We reviewed the medical records of the myopathy database from January 2002 to August 2021. We selected 6 muscle samples from 3 patients with Bethlem myopathy (MF226, MF1014, and MF1474) and 3 control subjects. Table 1 summarizes the clinical and genetic spectra of the study participants. Three patients with Bethlem myopathy showed typical clinical presentations, including proximal muscle weakness and multiple contractures, and were genetically confirmed. The variants carried by these 3 patients were pathogenic or likely pathogenic according to the American College of Medical Genetics and Genomics and the Association for Molecular Pathology guidelines,^[17]^ and have been previously reported as pathogenic variants.^[13,18]^ Three control subjects were selected from patients based on the following criteria: Psychogenic weakness; Normal muscle pathology; Normal serum creatine kinase level, and; Boys or girls under 20 years of age. The study was conducted following the declaration of Helsinki and institutional criteria. The research protocol was approved by the institutional review board of Gangnam Severance Hospital, Korea (IRB No: 3-2021-0300). Written informed consents were obtained from all participants. For participants below the age of 16, written informed consent were obtained from their parents.

**Table 1:** Clinical presentation of patients with Bethlem myopathy and control subjects.

Subject	Sex	Variant	Age at diagnosis, yr	Age at onset, yr	Inheritance	Clinical presentation	CK, IU/l	Muscle pathology
MF226	F	*COL6A2*: c.856-1G > C	10	6	Autosomal dominant	Prominent contractures of ankle and interphalangeal joints, and mild proximal muscle weakness	488	Increased internal nuclei, increased size variability, atrophic fibers, and a few degenerating fibers
MF1014	M	*COL6A1:* c.1056 + 2dup	18	12	Sporadic	Proximal muscle weakness and contractures of ankle and interphalangeal joints	432	Myopathic changes with degenerating fibers and interstitial fibrosis
MF1474	F	*COL6A1:* c.868G > C (p.G290R)	16	2	Autosomal dominant	Proximal muscle weakness, multiple contractures, and scoliosis	76	Increased fiber size variation, hypertrophic fibers, and type I predominance
Control 1	M	-	18	-	-	Psychogenic weakness	84	Normal finding
Control 2	M	-	14	-	-	Psychogenic weakness	137	Normal finding
Control 3	F	-	16	-	-	Psychogenic weakness	54	Normal finding

CK = creatine kinase.

### 2.2. RNA-seq

The total RNA concentration was calculated using the Quant-iT^TM^ Ribogreen RNA assay kit (Thermo Fisher Scientific, MA). To assess the integrity of the total RNA, 6 samples were run on a TapeStation RNA ScreenTape device (Agilent, Santa Clara, CA). Only high quality RNA preparations, with RNA integrity number values > 7.0, were used for RNA library construction. A library was prepared with 1 µg of total RNA from each sample using the Illumina TruSeq Stranded mRNA Sample Prep kit (Illumina, Inc., San Diego, CA). The first step in the workflow involves purifying the poly-A-containing mRNA molecules using poly-T oligo-attached magnetic beads. Following purification, mRNA was fragmented using divalent cations at elevated temperatures. The cleaved RNA fragments were copied into the first-strand complementary DNA (cDNA) using Superscript II reverse transcriptase (Thermo Fisher Scientific, MA), and random primers. This was followed by second-strand cDNA synthesis using DNA polymerase I, RNase H, and dUTP. These cDNA fragments then went through an end repair process, the addition of a single “A” base, and ligation of the indexing adapters. The products were purified and enriched using polymerase chain reaction (PCR) to create the final cDNA library. The libraries were quantified using quantitative PCR (qPCR) according to the qPCR Quantification Protocol Guide (KAPA Library Quantification kits for Illumina Sequencing platforms) and analyzed using the D1000 Screen Tape assay (Agilent Technologies, Waldbronn, Germany). Indexed libraries were sequenced on the NovaSeq 6000 platform (Illumina, San Diego, CA).

### 2.3. Statistical analysis of gene expression levels

The quality of the RNA-seq data was evaluated using Fast QC, and all were determined to be of high quality. The total number of reads was 112390858, 103286098, 121022280, 101810524, 109786544, and 118204882 in the skeletal muscles from MF226, MF1014, MF1474, control 1, control 2, and control 3, respectively. There were no significant differences in the sequencing depth or mapping efficiency between the 2 groups. We analyzed similarities and patterns among samples using hierarchical clustering and principal component analysis. Gene expression levels and volume plots were analyzed as previously described.^[19]^ Gene enrichment and functional annotation analysis for the significant genes was performed using gene ontology (GO) (www.geneontology.org/), and pathway analysis for the differentially expressed genes was performed based on the kyoto encyclopedia of genes and genomes (KEGG) pathways (http://www.genome.jp/kegg/pathway.html).

### 2.4. Availability of data and materials

The datasets generated and/or analyzed during the current study are deposited in the NCBI sequence read archive database (http://www.ncbi.nlm.nih.gov/bioproject/796623), under the accession numbers PRJNA796623.

## 3. Results

**Figure 1.**
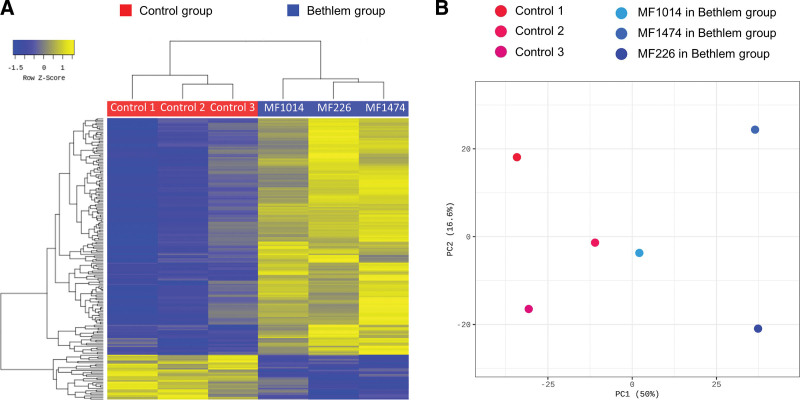
Heatmap for hierarchical clustering and principal component analysis (PCA) of the myopathy and control groups. (A) The heatmap for hierarchical clustering was generated using the R package “plots” using the expression for each gene (rows) and sample (columns). The expression levels of each gene across samples are shown as Z-scores scaled by their fragments per kilobase of transcript per million mapped reads (FPKMS) values from RNA-seq. The scaled expression values are color-coded according to the legend. The dendrogram depicting hierarchical clustering is based on the expression levels of all genes. (B) PCA of gene expression in skeletal muscles from the myopathy and control groups. Samples are color-coded according to the legend (blue circles: the Bethlem group; red circles: the control group). The numbers in blankets correspond to the proportion of variance explained by the respective principal component. RNA-seq = RNA-sequencing.

**Figure 2.**
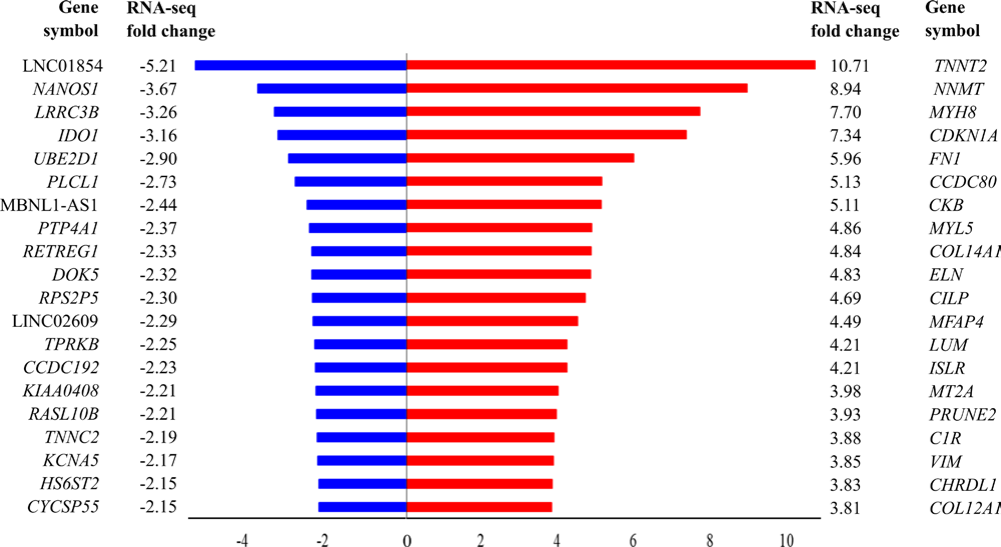
Top 20 genes that are significantly upregulated and downregulated with > 2-fold change.

The heat map for hierarchical clustering showed separation between the myopathy and control groups (Fig. 1A). Principal component analysis also showed a separate clustering of the samples by group (Fig. 1B). Among the 35,993 identified transcripts, 17,931 transcripts with 0 fragments per kilobase of transcripts per million fragments mapped were excluded. Of the remaining 18,062 transcripts with nonzero fragments per kilobase of transcripts per million, 187 transcripts were significantly differentially expressed (|fold change| ≥2, *P* < .05), with 157 upregulated and 30 downregulated transcripts in the Bethlem group (see Table S1, Supplemental Digital Content, http://links.lww.com/MD/I549, which represented the list of genes). A flow diagram for the identification of candidate genes is shown in Figure S1, Supplemental Digital Content, http://links.lww.com/MD/I550. The top 20 upregulated and downregulated genes are summarized in Figure 2. Among the noncoding RNAs, microRNA (miR)-133b was significantly upregulated, and 4 long intergenic non protein coding RNAs (lncRNAs), LINC01854, MBNL1-AS1, LINC02609, and LOC728975, were significantly downregulated.

The differentially expressed genes were categorized using GO. We identified 404 enriched GO terms in the Bethlem group versus the control group (false discovery rate-adjusted *P* < .05). The top 20 enriched GO terms came from categories centered on the ECM and are listed in Supplemental Digital Content (see Table S2, Supplemental Digital Content, http://links.lww.com/MD/I551). The KEGG pathway enrichment reflected themes with significant enrichment of the ECM-receptor interaction (has04512), complement and coagulation cascadehashsa04610), and focal adhehasn (hsa04510) in the Bethlem group (Table 2 and see Figure S2, Supplemental Digital Content, http://links.lww.com/MD/I552). Among the genes that mapped the complement and coagulation chasades (hsa04610), ten transcripts (*SERPING1, C1QA, C1QB, C1QC, C1R, C1S, C3, F3, F13A1*, and *CFH*) were significantly upregulated in the Bethlem group. Among the genes that mapped the ECM-receptor ihasraction (hsa04512), 7 transcripts (*CD44, COL6A1, COL6A3, FN1, HSPG2, THBS3*, and *TNXB*) were significantly upregulated in the Bethlem group (Table 3). *COL6A2* was also upregulated in the Bethlem group, but the difference was not statistically significant (*P* = .06). Three other collagen genes, *COL12A1, COL14A01*, and *COL16A1* were also significantly upregulated in the Bethlem group (Table 3).

**Table 2:** Kyoto encyclopedia of genes and genomes (KEGG) pathway analysis of genes commonly regulated in Bethlem myopathy.

Map ID	KEGG pathway	Genes	Rich factor* (%)	FDR
04610	Complement and coagulation cascades	*F3, F13A1, CFH, SERPING1, C1QA, C1QB, C1QC, C1R, C1S, C3*	11.76	1.66E–08
05133	Pertussis	*SERPING1, C1QA, C1QB, C1QC, C1R, C1S, C3, CD14*	10.53	2.76E–06
04510	Focal adhesion	*ACTB, ACTG1, BCL2, COL6A1. COL6A3, FN1, MYL5, PDGFRB, THBS3, TNXB*	4.98	5.90E–06
05205	Proteoglycans in cancer	*ACTB, ACTG1, CD44, CDKN1A, DCN, ERBB3, FN1, HSPG2, LUM, TIMP3*	4.88	5.90E–06
04974	Protein digestion and absorption	*ATP1A1, COL6A1, COL6A3, COL12A1, DPP4, ELN, COL14A1, COL16A1*	7.77	7.78E–06
05171	Coronavirus disease	*C1QA, C1QB, C1QC, C1R, C1S, C3, F13A1, RPL3, CCL2, STING1*	4.31	1.08E–05
04512	ECM-receptor interaction	*CD44, COL6A1, COL6A3, FN1, HSPG2, THBS3, TNXB*	7.95	4.21E–05
05150	*Staphylococcus aureus* infection	*C1QA, C1QB, C1QC, C1R, C1S, C3, CFH*	7.29	5.95E–05
05131	Shigellosis	*ACTB, ACTG1, BCL2, C3, CD14, CD44, MYL5, UBE2D1, STING1*	3.64	1.21E–04
05206	MicroRNAs in cancer	*BCL2, CD44, CDKN1A, ERBB3, PDGFRB, TIMP3, TNXB, VIM, MIR133B*	2.90	5.32E–04
04145	Phagosome	*ACTB, ACTG1, C1R, C3, CD14, THBS3, TUBA1A*	4.61	5.32E–04
05132	Salmonella infection	*ACTB, ACTG1, ANXA2, BCL2, CD14, MYL6B, S100A100, TUBA1A*	3.21	8.58E–04
04151	PI3K-Akt signalling pathway	*BCL2, CDKN1A, COL6A1, COL6A3, ERBB3, FN1, PDGFRB, THBS3, TNXB*	2.54	1.03E–03
05322	Systemic lupus erythematosus	*C1QA, C1QB, C1QC, C1R, C1S, C3*	4.41	2.67E–03
04810	Regulation of actin cytoskeleton	*ACTB, ACTG1, FN1, GSN, MYL5, PDGFRB, TMSB4X*	3.21	2.67E–03
05142	Chagas disease	*C1QA, C1QB, C1QC, C3, CCL2*	4.90	8.37E–03
04066	HIF-1 signalling pathway	*BCL2, CDKN1A, ENO2, LDHB, TIMP1*	4.59	1.00E–02
05144	Malaria	*ACKR1, LRP1, CCL2, THBS3*	8.00	1.12E–02
04978	Mineral absorption	*ATP1A1, MT1E, MT2A, CYBRD1*	6.67	1.69E–02
05165	Human papillomavirus infection	*CDKN1A, COL6A1, COL6A3, FN1, PDGFRB, THBS3, TNXB*	2.11	1.69E–02
05100	Bacterial invasion of epithelial cells	*ACTB, ACTG1, DNM1, FN1*	5.19	0.03
04530	Tight junction	*ACTB, ACTG1, TUBA1A, JAM3, MYL6B*	2.96	0.04

ECM = extracellular matrix, FDR = false discovery rate.

* The rich factor is the ratio of the number of differentially expressed genes (DEGs) to the background number in a certain pathway.

**Table 3:** Genes involved in the extracellular matrix (ECM) receptor interaction and other collagens that were significantly upregulated with > 2-fold change.

Gene symbol	Description	Individual FPKM for patients and % of control			Mean FPKM			
		MF226	MF1014	MF1474	Control (n = 3)	Patients (n = 3)	RNA-seq fold change	*P* value
Genes involved in ECM-receptor interaction								
* FN1*	Fibronectin 1	83.0	26.7	49.0	7.7	52.9	5.96	.032
* CD44*	CD44 molecule (Indian blood group)	23.5	11.7	21.4	3.6	18.9	3.50	.007
* TNXB*	Tenascin XB	27.7	22.6	21.7	5.7	24.0	3.28	.028
* COL6A1*	Collagen type VI alpha 1 chain	101.6	69.2	117.4	30.8	96.1	3.03	.023
* COL6A2*	Collagen type VI alpha 2 chain	112.3	94.7	199.2	48.1	135.4	2.93	.061
* COL6A3*	Collagen type VI alpha 3 chain	30.1	24.2	56.0	9.4	36.8	3.25	.036
* THBS3*	Thrombospondin 3	7.9	6.5	12.1	2.4	8.8	2.21	.019
* HSPG2*	Heparan sulfate proteoglycan 2	28.8	16.5	24.0	8.8	23.1	2.14	.046
Other collagen genes								
* COL12A1*	Collagen type XII alpha 1 chain	8.7	10.5	16.2	1.6	11.8	3.81	.008
* COL14A1*	Collagen type XIV alpha 1 chain	6.1	8.8	24.3	1.2	13.1	4.83	.042
* COL16A1*	Collagen type XVI alpha 1 chain	7.3	7.4	12.3	2.1	9.0	2.55	.033

ECM = extracellular matrix, FPKM = Fragments per kilobase of transcript per million mapped reads, RNA-seq = RNA-sequencing.

## 4. Discussion

Our results showed the gene expression profiles of skeletal muscles in patients with Bethlem myopathy. Bethlem myopathy was genetically confirmed, and the control subjects exhibited no clinical, pathological, or electrophysiological neuromuscular disorders. Although the sample size was small, skeletal muscles were obtained from both the myopathy and control group patients were teenagers. Therefore, our study was able to demonstrate the molecular changes in the skeletal muscles of patients with Bethlem myopathy.

Our study showed 157 upregulated and 30 downregulated transcripts in the Bethlem group. Among these, the upregulated transcripts were involved in the differentiation and regeneration of skeletal muscles, stabilizing muscle membranes, and major ECM components. In our study, the most significantly upregulated gene was *TNNT2*, which was associated with muscle regeneration. This is consistent with previous study findings.^[10]^ nicotinamide N-methyltransferase (NNMT) was the second most highly upregulated gene. The NNMT, encoded by *NNMT*, is an enzyme that is a major consumer of NAD^+^, involved in metabolic derangement in various tissues.^[20]^ Under normal conditions, NNMT is expressed mainly in the liver and skeletal muscles. Dysregulation of NNMT leads to reduced expression of nicotinamide phosphoribosyl transferase, a major enzyme involved in NAD^+^ biosynthesis. Since the cofactor NAD^+^ plays an important role in stabilizing muscles from metabolic and structural degeneration, *NNMT* is upregulated in Duchene muscular dystrophy, other muscle diseases, and *mdx* mice.^[21]^ Among the noncoding RNAs, miR-133b was significantly upregulated in the Bethlem group. miR-133b promotes fibrosis in renal cells and is associated with chronic chagas disease and dilated cardiomyopathy.^[22,23]^ One study previously suggested that noncoding RNAs, miR-181a and miR-30c, may play an important role in regulating gene expression in patients with Ullrich congenital muscular dystrophy; however, there has been no evaluation of miR-133b so far.^[9]^ In the present study, miR-181a and miR-30c were not significantly expressed.

Our results also revealed several downregulated transcripts. *NANOS1* was the most significantly downregulated protein coding gene. *NANOS1* controls cell cycle progression and affects the SMAD Family Member 3/transforming growth factor (TGF)-β fibroblast maturation pathway.^[24,25]^ One group previously identified significant GO categories of downregulated genes associated with posttranscriptional regulation of gene expression in patients with Ullrich congenital muscular dystrophy compared with controls, among which *NANOS1* was identified.^[10]^ The second most downregulated protein coding gene was *LRRC3B*, which is a tumor suppressor.^[26]^ Indoleamine 2, 3-dioxygenase, encoded by *IDO1*, catalyzes the first and rate-limiting step in tryptophan catabolism to kynurenine, and plays an important role in tumor cell evasion of the immune system.^[27]^ lncRNAs are known to play essential roles in the proliferation and apoptosis of cells, particularly cancer cells.^[28]^ Our study showed that these 4 lncRNAs were significantly downregulated. LINC02609 was previously reported as a key lncRNA associated with distant metastasis and poor prognosis in patients with clear cell renal cell carcinoma.^[29]^ MBNL1-AS1 is also known to repress proliferation and enhance apoptosis in bladder cancer cells.^[30]^ However, our results could not be confirmed by previous studies.

The KEGG pathway analysis showed significant enrichment of the ECM-receptor interaction (hsa04512), complement and coagulation cascades (hsa04610), and focal adhesion (hsa04510) in the Bethlem group. The ECM-receptor interaction pathway has been shown to be significantly differentially expressed in many studies.^[7,9–11]^ However, the 3 collagen-6-related genes (*COL6A1, COL6A2*, and *COL6A3*) were highly elevated in the Bethlem group, regardless of whether the mechanism of variants had a loss of function or a dominant-negative effect. This is contrary to the results of a previous study that showed that the expression of *COL6A1, COL6A2*, and *COL6A3* depends on the inheritance mechanism.^[31]^ It is known that the complement and coagulation cascades (hsa04610) and focal adhesion (hsa04510) are linked to wound healing, and that collagen VI plays an important role in this process.^[32,33]^ The relationship between collagen VI-related myopathy and wound healing has been previously reported in dermal fibroblasts from patients with Ullrich congenital muscular dystrophy.^[9]^ However, our results did not identify differences in several previously reported pathways, including the cell cycle (hsa04110), DNA replication (hsa03030), nitrogen metabolism (hsa00910), TGF-beta signaling pathway (hsa04350), arrhythmogenic right ventricular cardiomyopathy (hsa05412), hypertrophic cardiomyopathy (hsa05410), viral myocarditis (hsa05416), hematopoietic cell lineage (hsa04640), renin-angiotensin system (hsa04614), or circadian rhythm (hsa04710).^[9,11,31,34]^ Among them, the TGF-β signaling pathway (hsa04350) showed significant changes in several studies.^[9,11,31,34]^ The difference between the results of this study and those of previous studies can be attributed to a variety of reasons. First, most studies, including ours, were performed with a small number of samples. Second, the subject specimens were diverse, such as the patients muscles, cultured fibroblasts, and mouse model muscles. Third, patient age and disease severity may have influenced the results.

Our study has several limitations. The major limitation was the small number of muscle samples from the patients with Bethlem myopathy and controls. Second, we could not use age- or sex-matched controls. If the number of patients with Bethlem myopathy is large to allow studies to subgroup them according to age, sex, clinical severity, mutation type, and we could further reinforce and complement our results.

## 5. Conclusion

In conclusion, we confirmed that Bethlem myopathy is strongly associated with the production and organization of ECM and the wound healing process. Additionally, our results suggest that the pathogenesis of Bethlem myopathy is influenced by several nonprotein-coding genes.

## Acknowledgments

The authors would like to thank the patients for their help and involvement in this study.

## Author contributions

**Conceptualization:** Seung-Ah Lee, Hyung Jun Park, Young-Chul Choi.

**Data curation:** Ji-Man Hong.

**Formal analysis:** Jung Hwan Lee.

**Software:** Hyung Jun Park.

**Supervision:** Young-Chul Choi.

**Validation:** Seung-Ah Lee, Ji-Man Hong.

**Writing – original draft:** Seung-Ah Lee, Jung Hwan Lee.

**Writing – review & editing:** Seung-Ah Lee, Hyung Jun Park.

## Supplementary Material








