# Supplementary material for: Transcriptome profiling of skeletal muscles from Korean patients with Bethlem myopathy
Source: Medicine (Baltimore). 2023 Mar 3;102(9):e33122. doi: 10.1097/MD.0000000000033122 (PMC9981387; doi:10.1097/MD.0000000000033122)
Supplement: Supplementary file 1 [file medi-102-e33122-s001.pdf]

Supplementary Table 1. Significantly upregulated and downregulated genes with more than two-fold change

Gene symbol	Description	RNA-seq fold change
TNNT2	troponin T2, cardiac type	10.71
NNMT	nicotinamide N-methyltransferase	8.94
MYH8	myosin heavy chain 8	7.70
CDKN1A	cyclin dependent kinase inhibitor 1A	7.34
FN1	fibronectin 1	5.96
CCDC80	coiled-coil domain containing 80	5.12
CKB	creatine kinase B	5.11
MYL5	myosin light chain 5	4.86
COL14A1	collagen type XIV alpha 1 chain	4.84
ELN	elastin	4.83
CILP	cartilage intermediate layer protein	4.69
MFAP4	microfibril associated protein 4	4.49
LUM	lumican	4.21
ISLR	immunoglobulin superfamily containing leucine rich repeat	4.21
MT2A	metallothionein 2A	3.98
PRUNE2	prune homolog 2 with BCH domain	3.93
C1R	complement C1r	3.88
VIM	vimentin	3.85
CHRDL1	chordin like 1	3.83
COL12A1	collagen type XII alpha 1 chain	3.81
EEF1A1	eukaryotic translation elongation factor 1 alpha 1	3.81
MYL6B	myosin light chain 6B	3.78
S100A10	S100 calcium binding protein A10	3.77
S100A6	S100 calcium binding protein A6	3.76
PLTP	phospholipid transfer protein	3.75
PODN	podocan	3.75
TIMP1	TIMP metallopeptidase inhibitor 1	3.72
CCL14	C-C motif chemokine ligand 14	3.67
MIR133B	microRNA 133b	3.62
ADAMTSL4	ADAMTS like 4	3.61
EMP3	epithelial membrane protein 3	3.56
CD14	CD14 molecule	3.54
EEF1A1P6	eukaryotic translation elongation factor 1 alpha 1 pseudogene 6	3.51
CRABP2	cellular retinoic acid binding protein 2	3.51
FBLN1	fibulin 1	3.50
CD44	CD44 molecule (Indian blood group)	3.50
ACTG1	actin gamma 1	3.48
ANXA1	annexin A1	3.47
LGALS3BP	galectin 3 binding protein	3.43
THRSP	thyroid hormone responsive	3.42
EEF1A1P5	eukaryotic translation elongation factor 1 alpha 1 pseudogene 5	3.40
CRIP1	cysteine rich protein 1	3.34
TNXB	tenascin XB	3.28
TNXA	tenascin XA (pseudogene)	3.25
COL6A3	collagen type VI alpha 3 chain	3.25
ADIRF	adipogenesis regulatory factor	3.23
TIMP2	TIMP metallopeptidase inhibitor 2	3.22
GSN	gelsolin	3.20

C1QA	complement C1q A chain	3.20
EFEMP1	EGF containing fibulin extracellular matrix protein 1	3.15
CFH	complement factor H	3.11
ERBB3	erb-b2 receptor tyrosine kinase 3	3.08
PAMR1	peptidase domain containing associated with muscle regeneration 1	3.03
COL6A1	collagen type VI alpha 1 chain	3.03
MXRA8	matrix remodeling associated 8	3.02
SNX7	sorting nexin 7	3.02
TUBA1A	tubulin alpha 1a	3.00
C1S	complement C1s	2.98
C14orf132	chromosome 14 open reading frame 132	2.94
TMEM176B	transmembrane protein 176B	2.93
ERRFI1	ERBB receptor feedback inhibitor 1	2.91
ANXA2	annexin A2	2.88
TMEM176A	transmembrane protein 176A	2.87
PLAC9	placenta associated 9	2.87
C3	complement C3	2.85
C1QB	complement C1q B chain	2.84
SERPING1	serpin family G member 1	2.84
OAF	out at first homolog	2.80
BASP1	brain abundant membrane attached signal protein 1	2.79
NES	nestin	2.77
ABLIM1	actin binding LIM protein 1	2.75
LSP1	lymphocyte specific protein 1	2.74
DCN	decorin	2.72
GALNT17	polypeptide N-acetylgalactosaminyltransferase 17	2.68
C1QC	complement C1q C chain	2.64
TAGLN2	transgelin 2	2.64
PNMA8A	PNMA family member 8A	2.64
RPL3	ribosomal protein L3	2.63
ATP1A1	ATPase Na+/K+ transporting subunit alpha 1	2.63
SIK1	salt inducible kinase 1	2.61
IFI27L2	interferon alpha inducible protein 27 like 2	2.59
CYBRD1	cytochrome b reductase 1	2.58
ANGPTL2	angiopoietin like 2	2.56
COL16A1	collagen type XVI alpha 1 chain	2.55
MVP	major vault protein	2.54
LOXL1	lysyl oxidase like 1	2.52
TMSB4X	thymosin beta 4 X-linked	2.51
GPNMB	glycoprotein nmb	2.49
TNFAIP2	TNF alpha induced protein 2	2.49
DDAH2	dimethylarginine dimethylaminohydrolase 2	2.48
PCDHGC3	protocadherin gamma subfamily C, 3	2.47
SRPX	sushi repeat containing protein X-linked	2.47
PLXNB2	plexin B2	2.45
CNN3	calponin 3	2.45
HSD3B7	hydroxy-delta-5-steroid dehydrogenase, 3 beta- and steroid delta-isomerase 7	2.42
ADAM33	ADAM metallopeptidase domain 33	2.39
LDHB	lactate dehydrogenase B	2.37
EPB41L3	erythrocyte membrane protein band 4.1 like 3	2.37
EPS8L2	EPS8 like 2	2.37
OSR1	odd-skipped related transcription factor 1	2.37

LTBP2	latent transforming growth factor beta binding protein 2	2.37
ECM1	extracellular matrix protein 1	2.37
LRP1	LDL receptor related protein 1	2.37
PPIA	peptidylprolyl isomerase A	2.35
ENO2	enolase 2	2.33
TSKU	tsukushi, small leucine rich proteoglycan	2.32
VAT1	vesicle amine transport 1	2.32
MATN2	matrilin 2	2.29
RARRES2	retinoic acid receptor responder 2	2.29
DNM1	dynamin 1	2.29
MT1E	metallothionein 1E	2.28
ID3	inhibitor of DNA binding 3, HLH protein	2.28
RAB34	RAB34, member RAS oncogene family	2.26
F13A1	coagulation factor XIII A chain	2.26
CHRND	cholinergic receptor nicotinic delta subunit	2.24
DMPK	DM1 protein kinase	2.23
SELENOM	selenoprotein M	2.23
ABHD2	abhydrolase domain containing 2	2.23
THBS3	thrombospondin 3	2.21
JAM3	junctional adhesion molecule 3	2.20
ZFP36L1	ZFP36 ring finger protein like 1	2.20
DBN1	drebrin 1	2.20
DPP4	dipeptidyl peptidase 4	2.20
LOXL4	lysyl oxidase like 4	2.18
STING1	stimulator of interferon response cGAMP interactor 1	2.18
TSPAN4	tetraspanin 4	2.16
NRBP2	nuclear receptor binding protein 2	2.15
IFITM1	interferon induced transmembrane protein 1	2.15
TNFAIP8L3	TNF alpha induced protein 8 like 3	2.14
PPP1R18	protein phosphatase 1 regulatory subunit 18	2.14
HSPG2	heparan sulfate proteoglycan 2	2.14
DAB2	DAB adaptor protein 2	2.13
MEDAG	mesenteric estrogen dependent adipogenesis	2.13
F3	coagulation factor III, tissue factor	2.12
CCL2	C-C motif chemokine ligand 2	2.12
FOLR2	folate receptor beta	2.10
CASQ2	calsequestrin 2	2.10
OLFML3	olfactomedin like 3	2.09
HMCN2	hemicentin 2	2.08
ANXA5	annexin A5	2.08
PDGFRB	platelet derived growth factor receptor beta	2.08
MARVELD1	MARVEL domain containing 1	2.08
VWA5A	von Willebrand factor A domain containing 5A	2.08
LGALS3	galectin 3	2.07
ACKR1	atypical chemokine receptor 1 (Duffy blood group)	2.07
CHPF	chondroitin polymerizing factor	2.07
LSP1P4	LSP1 pseudogene 4	2.06
TTLL3	tubulin tyrosine ligase like 3	2.05
ACTB	actin beta	2.05
XG	Xg glycoprotein (Xg blood group)	2.04
GOLGA8A	golgin A8 family member A	2.04
SFXN3	sideroflexin 3	2.03
APOBEC3C	apolipoprotein B mRNA editing enzyme catalytic subunit 3C	2.03
STAT6	signal transducer and activator of transcription 6	2.01

ABR	ABR activator of RhoGEF and GTPase	2.00
TIMP3	TIMP metallopeptidase inhibitor 3	2.00
MIR6845	hsa-miR-6845-3p	2.00
ARMCX5-GPRASP2	ARMCX5-GPRASP2 readthrough	-2.00
BCL2	BCL2 apoptosis regulator	-2.01
TRIM63	tripartite motif containing 63	-2.02
WWP1	WW domain containing E3 ubiquitin protein ligase 1	-2.04
CA4	carbonic anhydrase 4	-2.04
RNF128	ring finger protein 128	-2.06
FBXL17	F-box and leucine rich repeat protein 17	-2.06
NDUFA5	NADH dehydrogenase 1alpha subcomplex subunit 5	-2.07
GCSHP5	glycine cleavage system protein H pseudogene 5	-2.09
LOC728975	uncharacterized LOC728975	-2.12
CYCSP55	CYCS pseudogene 55	-2.15
HS6ST2	heparan sulfate 6-O-sulfotransferase 2	-2.15
KCNA5	potassium voltage-gated channel subfamily A member 5	-2.17
TNNC2	troponin C2, fast skeletal type	-2.19
RASL10B	RAS like family 10 member B	-2.21
KIAA0408	KIAA0408	-2.21
CCDC192	coiled-coil domain containing 192	-2.23
TPRKB	TP53RK binding protein	-2.25
LINC02609	long intergenic non-protein coding RNA 2609	-2.29
RPS2P5	ribosomal protein S2 pseudogene 5	-2.30
DOK5	docking protein 5	-2.32
RETREG1	reticulophagy regulator 1	-2.33
PTP4A1	protein tyrosine phosphatase 4A1	-2.37
MBNL1-AS1	MBNL1 antisense RNA 1	-2.44
PLCL1	phospholipase C like 1 (inactive)	-2.73
UBE2D1	ubiquitin conjugating enzyme E2 D1	-2.90
IDO1	indoleamine 2,3-dioxygenase 1	-3.16
LRRC3B	leucine rich repeat containing 3B	-3.26
NANOS1	nanos C2HC-type zinc finger 1	-3.67
LINC01854	long intergenic non-protein coding RNA 1854	-5.21
