# Supplementary material for: Transcriptome profiling of skeletal muscles from Korean patients with Bethlem myopathy
Source: Medicine (Baltimore). 2023 Mar 3;102(9):e33122. doi: 10.1097/MD.0000000000033122 (PMC9981387; doi:10.1097/MD.0000000000033122)

Supplementary Figure 1. Flow diagram for the identification of candidate genes in Bethlem myopathy

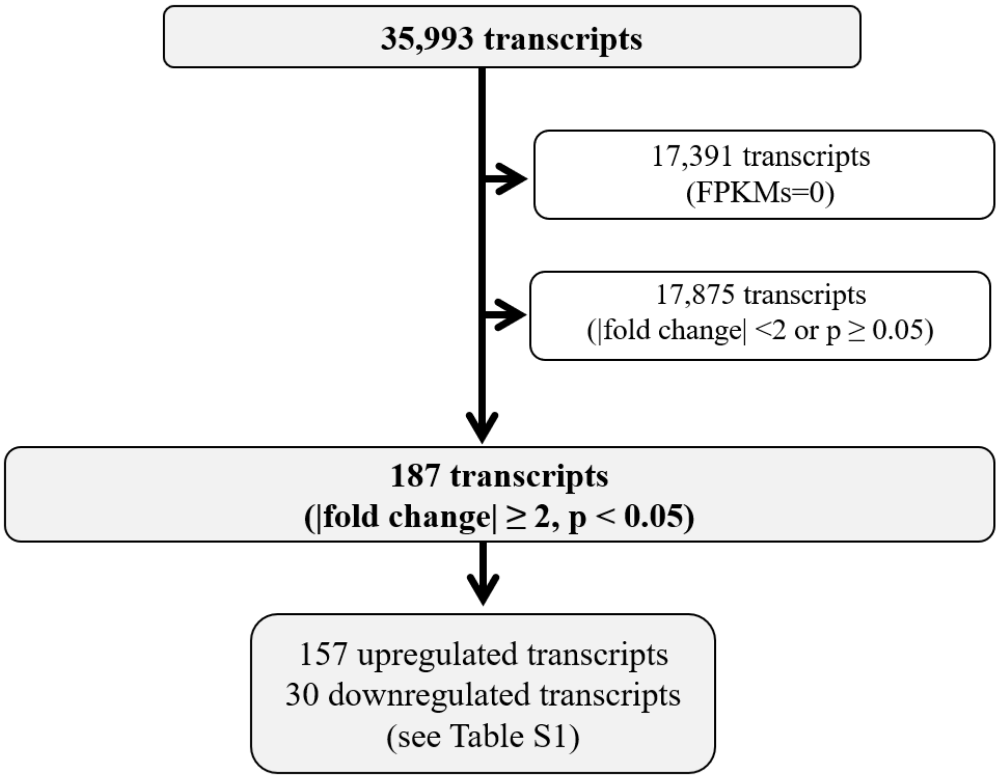

Supplement: Supplementary file 2 [file medi-102-e33122-s002.pdf]
