# Supplementary material for: Transcriptome profiling of skeletal muscles from Korean patients with Bethlem myopathy
Source: Medicine (Baltimore). 2023 Mar 3;102(9):e33122. doi: 10.1097/MD.0000000000033122 (PMC9981387; doi:10.1097/MD.0000000000033122)
Supplement: Supplementary file 3 [file medi-102-e33122-s003.pdf]

Supplementary Table 2. Gene ontology enrichment analysis of differentially expressed genes associated with Collagen VI-related myopathy

GO term	Description	*Category	†Rich factor (%)	p-valve
0062023	collagen-containing extracellular matrix	CC	9.98	1.23E-29
0031012	extracellular matrix	CC	7.65	4.57E-26
0030312	external encapsulating structure	CC	7.64	4.57E-26
0005201	extracellular matrix structural constituent	MF	12.72	1.56E-16
0005576	extracellular region	CC	2.04	2.78E-16
0005615	extracellular space	CC	2.25	5.78E-16
0043230	extracellular organelle	CC	2.60	1.90E-13
1903561	extracellular vesicle	CC	2.61	1.90E-13
0070062	extracellular exosome	CC	2.66	1.90E-13
0065010	extracellular membrane-bounded organelle	CC	2.60	1.90E-13
0005198	structural molecule activity	MF	4.56	6.94E-12
0031982	vesicle	CC	1.87	1.68E-10
0030198	extracellular matrix organization	BP	5.73	9.68E-10
0043062	extracellular structure organization	BP	5.71	9.68E-10
0045229	external encapsulating structure organization	BP	5.69	9.68E-10
0034774	secretory granule lumen	CC	5.63	2.16E-08
0060205	cytoplasmic vesicle lumen	CC	5.56	2.41E-08
0031983	vesicle lumen	CC	5.52	2.46E-08
0005581	collagen trimer	CC	10.53	3.80E-07
0062023	collagen-containing extracellular matrix	CC	9.98	1.23E-29

GO, gene ontology; FDR, False Discovery Rate

*Category: BP, biological process; BP, biological process; CC, cellular component,

†Rich factor is the ratio of differentially expressed gene numbers annotated in the pathway term to all gene numbers annotated
