# Supplementary figures and images for: Transcriptome profiling of skeletal muscles from Korean patients with Bethlem myopathy
Source: Medicine (Baltimore). 2023 Mar 3;102(9):e33122. doi: 10.1097/MD.0000000000033122 (PMC9981387; doi:10.1097/MD.0000000000033122)

(A)

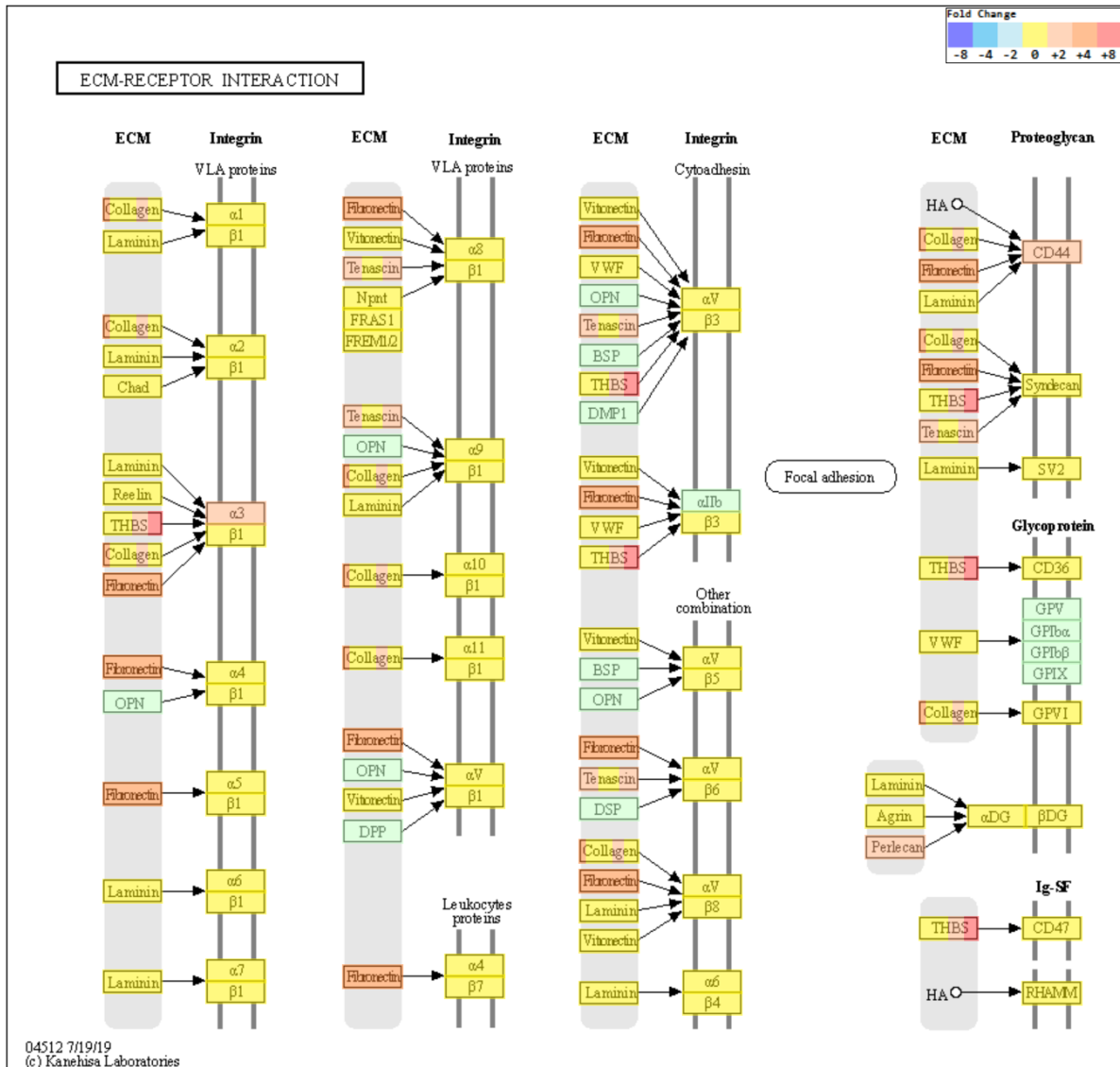

(B)

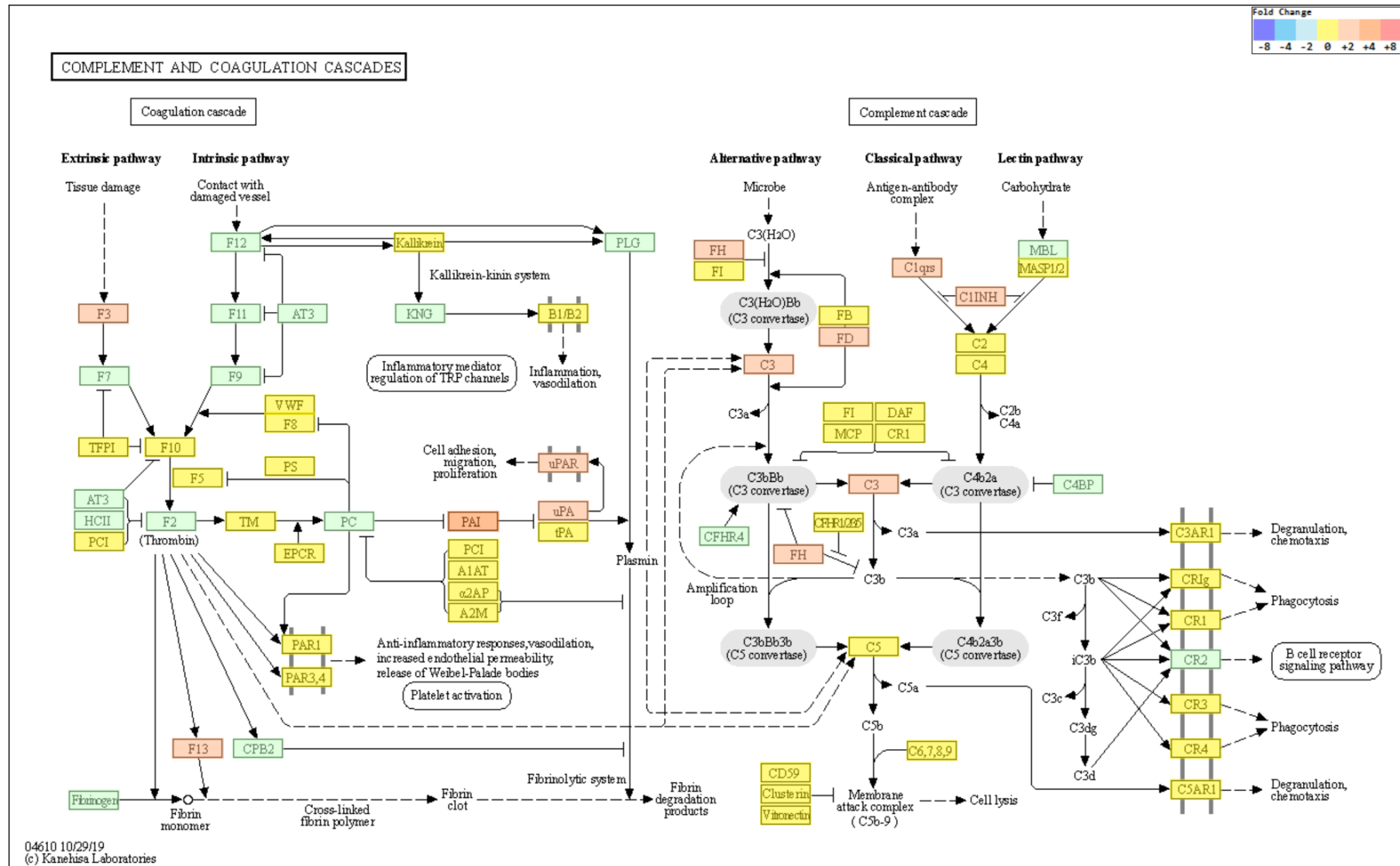

(C)

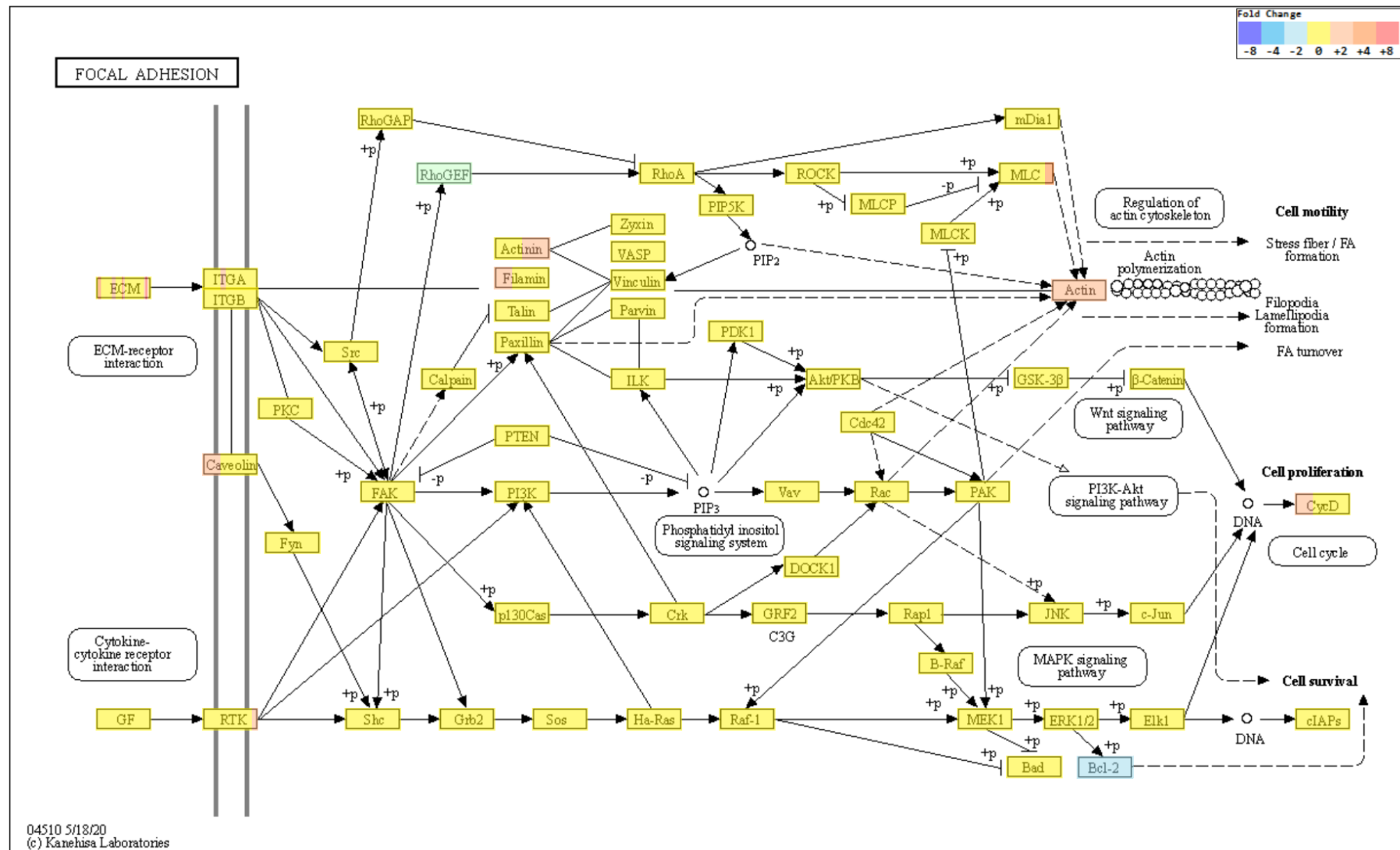

Supplement: Supplementary file 4 [file medi-102-e33122-s004.pdf]
